# Comorbidity prevalence among cancer patients: a population-based cohort study of four cancers

**DOI:** 10.1186/s12885-019-6472-9

**Published:** 2020-01-28

**Authors:** Helen Fowler, Aurelien Belot, Libby Ellis, Camille Maringe, Miguel Angel Luque-Fernandez, Edmund Njeru Njagi, Neal Navani, Diana Sarfati, Bernard Rachet

**Affiliations:** 1 Cancer Survival Group, Department of Non-Communicable Disease Epidemiology, London School of Hygiene & Tropical Medicine, Keppel Street, London, WC1E 7HT UK; 2 Biomedical Research Institute of Granada, Non-Communicable and Cancer Epidemiology Group, University of Granada, Granada, Spain; 3 Department of Non-Communicable Disease Epidemiology, London School of Hygiene & Tropical Medicine, London, UK; 4 UCL Respiratory, University College London, London, UK; 5 Department of Thoracic Medicine, University College London Hospital, London, UK; 6 Department of Public Health, University of Otago, Wellington, New Zealand

**Keywords:** Cancer, Comorbidity, Multimorbidity, Deprivation, Prevalence, England, Epidemiology

## Abstract

**Background:**

The presence of comorbidity affects the care of cancer patients, many of whom are living with multiple comorbidities. The prevalence of cancer comorbidity, beyond summary metrics, is not well known. This study aims to estimate the prevalence of comorbid conditions among cancer patients in England, and describe the association between cancer comorbidity and socio-economic position, using population-based electronic health records.

**Methods:**

We linked England cancer registry records of patients diagnosed with cancer of the colon, rectum, lung or Hodgkin lymphoma between 2009 and 2013, with hospital admissions records. A comorbidity was any one of fourteen specific conditions, diagnosed during hospital admission up to 6 years prior to cancer diagnosis. We calculated the crude and age-sex adjusted prevalence of each condition, the frequency of multiple comorbidity combinations, and used logistic regression and multinomial logistic regression to estimate the adjusted odds of having each condition and the probability of having each condition as a single or one of multiple comorbidities, respectively, by cancer type.

**Results:**

Comorbidity was most prevalent in patients with lung cancer and least prevalent in Hodgkin lymphoma patients. Up to two-thirds of patients within each of the four cancer patient cohorts we studied had at least one comorbidity, and around half of the comorbid patients had multiple comorbidities. Our study highlighted common comorbid conditions among the cancer patient cohorts. In all four cohorts, the odds of having a comorbidity and the probability of multiple comorbidity were consistently highest in the most deprived cancer patients.

**Conclusions:**

Cancer healthcare guidelines may need to consider prominent comorbid conditions, particularly to benefit the prognosis of the most deprived patients who carry the greater burden of comorbidity. Insight into patterns of cancer comorbidity may inform further research into the influence of specific comorbidities on socio-economic inequalities in receipt of cancer treatment and in short-term mortality.

## Background

Comorbidity refers to the existence of a long-term health condition in the presence of a primary disease of interest [1]. Having one or more comorbidities may influence the patient’s prognosis for a primary disease such as cancer. Comorbidity may influence the timing of cancer diagnosis, in either a positive or a negative way. For example, the symptoms of comorbidity may drive a patient to seek medical care sooner, potentially leading to an earlier diagnosis. Alternatively, cancer symptoms may be mistakenly considered as symptoms of a pre-existing health condition, and could delay diagnosis [2–4]. Following diagnosis, the presence of comorbidity may also influence timing, receipt, or outcome of treatment, with clear evidence that those with comorbidity are less likely to receive curative treatment than those without, despite increasing evidence that many patients with comorbidity benefit from such treatment [3]. Although the presence of multiple co-existent health conditions is commonplace, the guidelines, funding and structures of primary care may not support the care of more patients with multiple conditions [5], and care in secondary and tertiary centres is typically highly siloed [3].

Methods used in the scientific literature to describe, measure and quantify the status of comorbidity as an explanatory factor in adverse disease outcomes are varied. Many summarised metrics of comorbidity have been proposed, providing an overall picture of a patient’s comorbidity status, some specific to a primary disease while others are more general. For example, a widely used metric of comorbidity in epidemiological studies is the Charlson Comorbidity Index (CCI) [6], which weights 19 long-term health conditions according to their relative risk of one-year mortality, to produce an overall index score.

In this study, we firstly aimed to examine the prevalence of comorbid conditions in cancer patients using English population-based electronic health records of patients diagnosed with cancer of the colon, rectum, or lung or with Hodgkin lymphoma (HL). An association between comorbidity (not specific to any primary disease of interest) and socio-economic position has been widely reported: the prevalence of certain specific comorbid conditions [7–10] and general comorbidity prevalence being higher in deprived groups of patients [11–13]. Our second aim was to describe patterns of comorbidities and multiple comorbidity in these cancer patient cohorts, according to patient characteristics such as socio-economic position (deprivation).

## Methods

We defined a comorbid condition as one of the following fourteen health conditions: myocardial infarction (MI), congestive heart failure (CHF), peripheral vascular disease (PVD), cerebrovascular disease (CVD), dementia, chronic obstructive pulmonary disease (COPD), rheumatological conditions, liver disease, diabetes, hemiplegia or paraplegia, renal disease, previous malignancy, obesity or hypertension. The conditions, selected following a systematic search of the data, included conditions of the Charlson Comorbidity Index [6] and any highly prevalent conditions that may influence cancer management alone or in combination with another condition.

### Data

This study used England National Cancer Registry data of 331,655 patients aged 15–90 years at diagnosis with cancer of the colon, rectum, lung or Hodgkin’s lymphoma, between 2009 and 2013. Registry data provided information on patient sex, age at diagnosis, site of cancer, date of cancer diagnosis and area of residence at time of diagnosis, which was used to derive socio-economic position, based on deprivation quintiles of the Income Domain of the Indices of Multiple Deprivation [14]. The five-level, ordinal variable indicates the level of deprivation from 1 (least deprived) to 5 (most deprived). Areas of residence are defined at the Lower Super Output Area level (mean population 1500).

Inpatient, outpatient and emergency hospital admissions records (Hospital Episode Statistics, HES) [15] were successfully linked with over 99% of the cancer registry records, using common unique variables present in both data sources. The International Statistical Classification of Diseases and Related Health Conditions tenth edition (ICD-10) [16] codes captured within the diagnostic fields of HES records provided information on health conditions recorded during hospital admissions. We used the ICD-10 code groupings of health conditions proposed by Quan and colleagues for defining comorbidities using administrative data (see Additional file 1) [17], and used an algorithm [18] to identify whether these conditions had been recorded in the six-year period prior to cancer diagnosis. In contrast to the approach of Maringe and colleagues [18], we included diagnoses of conditions recorded up to 6 months prior to cancer diagnosis. We anticipated that first-time diagnoses of the conditions could occur in this period, and wanted to obtain the most complete picture of patient comorbidity. We used cancer registry data to identify whether a patient had been diagnosed with an unrelated malignancy up to 6 years before their diagnosis with the cancer of interest.

### Descriptive data analysis

We calculated the prevalence of a comorbid condition within each of the four patient cohorts defined by cancer site, firstly as a crude measure, calculating the percentage of patients who had a recorded diagnosis of the comorbidity in HES records, and secondly adjusting for age and sex to account for the older age demographic of cancer patient populations. Weights for this adjustment were obtained from 2011 UK census published population estimates of persons living in England [19].

### Statistical analysis

Logistic regression models were used to estimate the odds ratio (OR) of having each comorbidity by cancer site, adjusting for sex, age at cancer diagnosis and deprivation group. The binary outcome variable indicated the presence of the comorbidity. To account for a non-linear association between increasing age and the presence of comorbidity, age was modelled as a continuous variable using a restricted cubic spline with one knot fixed at 70 years in analyses conducted for cancers of the colon, rectum and lung and at 45 years for HL (the knot position was chosen as to be close to the mean age of the patients in each of these cancer cohorts). To reduce the risk of unstable models, we ensured there were at least ten or more occurrences of a comorbidity within the specific cancer patient cohort for every parameter of the model (events per variable, EPV) [20].

Multinomial logistic regression was used to estimate the probability of having a given comorbidity, either in isolation, or as one of multiple comorbidities, according to cancer site. The three-category outcome variable indicated whether the patient did not have the given comorbidity, only had this comorbidity, or had this comorbidity with other comorbidities. Models were adjusted for age, sex and deprivation, and were run for each cancer site and comorbidity combination with at least ten EPV.

All data analyses were conducted in STATA v.15.1 [21].

## Results

### Patient characteristics

The characteristics of patients diagnosed with cancer of the colon (*N* = 102,216), rectum (*N* = 56,342), lung (*N* = 165,677) or with HL (*N* = 7420) between 2009 and 2013, stratified by comorbidity status, are shown in Table 1. The majority of patients in each cohort were male: approximately 55% of colon, lung and HL patients and 63% of rectal cancer patients. At least 80% of colon, rectum and lung cancer patients were in the two oldest age group categories, while 50% of the HL patients were within the two youngest age groups. There was an even distribution of patients among each of the deprivation groups, except among lung cancer patients, where the percentage of patients in each group increased with deprivation level.

**Table 1:** Patient characteristics according to comorbidity status, by cancer

	Cancer																															
	Colon								Rectum								Lung								Hodgkin lymphoma							
	All patients		Number of patient comorbidities						All patients		Number of patient comorbidities						All patients		Number of patient comorbidities						All patients		Number of patient comorbidities					
			0		1		2+				0		1		2+				0		1		2+				0		1		2+	
	N	%	n	%	n	%	n	%	N	%	n	%	n	%	n	%	N	%	n	%	n	%	n	%	N	%	n	%	n	%	n	%
Sex																																
Male	54,425	53.2	23,455	43.1	14,887	27.4	16,083	29.6	35,630	63.2	18,782	52.7	8850	24.8	7998	22.4	91,568	55.3	29,333	32.0	25,283	27.6	36,952	40.4	4163	56.1	2907	69.8	697	16.7	559	13.4
Female	47,791	46.8	21,339	44.7	13,878	29.0	12,574	26.3	20,712	36.8	11,044	53.3	5299	25.6	4369	21.1	74,109	44.7	24,661	33.3	21,536	29.1	27,912	37.7	3257	43.9	2307	70.8	573	17.6	377	11.6
Age at cancer diagnosis (years)																																
15–29	769	0.8	661	86.0	103	13.4	5	0.7	207	0.4	180	87.0	22	10.6	5	2.4	178	0.1	120	67.4	52	29.2	6	3.4	2111	28.5	1885	89.3	205	9.7	21	1.0
30–44	2666	2.6	2151	80.7	416	15.6	99	3.7	1552	2.8	1302	83.9	203	13.1	47	3.0	1757	1.1	1212	69.0	435	24.8	110	6.3	1660	22.4	1412	85.1	194	11.7	54	3.3
45–59	11,971	11.7	8035	67.1	2619	21.9	1317	11.0	9597	17.0	7143	74.4	1635	17.0	819	8.5	19,923	12.0	10,768	54.0	5574	28.0	3581	18.0	1461	19.7	1001	68.5	288	19.7	172	11.8
60–74	42,166	41.3	20,696	49.1	11,696	27.7	9774	23.2	25,230	44.8	14,073	55.8	6421	25.4	4736	18.8	75,085	45.3	25,973	34.6	22,241	29.6	26,871	35.8	1398	18.8	651	46.6	366	26.2	381	27.3
75–90	44,644	43.7	13,251	29.7	13,931	31.2	17,462	39.1	19,756	35.1	7128	36.1	5868	29.7	6760	34.2	68,734	41.5	15,921	23.2	18,517	26.9	34,296	49.9	790	10.6	265	33.5	217	27.5	308	39.0
Deprivation group (IMD income)																																
Least deprived	22,411	21.9	10,864	48.5	6331	28.2	5216	23.3	11,879	21.1	6839	57.6	2939	24.7	2101	17.7	23,066	13.9	8589	37.2	6528	28.3	7949	34.5	1339	18.0	980	73.2	217	16.2	142	10.6
2	22,623	22.1	10,484	46.3	6303	27.9	5836	25.8	12,222	21.7	6810	55.7	3031	24.8	2381	19.5	28,411	17.1	9913	34.9	8025	28.2	10,473	36.9	1428	19.2	1013	70.9	222	15.5	193	13.5
3	21,591	21.1	9460	43.8	6123	28.4	6008	27.8	11,750	20.9	6219	52.9	2970	25.3	2561	21.8	32,822	19.8	10,980	33.5	9365	28.5	12,477	38.0	1462	19.7	1018	69.6	263	18.0	181	12.4
4	19,940	19.5	8118	40.7	5614	28.2	6208	31.1	11,266	20.0	5646	50.1	2838	25.2	2782	24.7	39,220	23.7	12,356	31.5	10,885	27.8	15,979	40.7	1618	21.8	1132	70.0	277	17.1	209	12.9
Most deprived	15,651	15.3	5868	37.5	4394	28.1	5389	34.4	9225	16.4	4312	46.7	2371	25.7	2542	27.6	42,158	25.4	12,156	28.8	12,016	28.5	17,986	42.7	1573	21.2	1071	68.1	291	18.5	211	13.4
TOTAL	102,216	100.0	44,794	43.8	28,765	28.1	28,657	28.0	56,342	100.0	29,826	52.9	14,149	25.1	12,367	21.9	165,677	100.0	53,994	32.6	46,819	28.3	64,864	39.2	7420	100.0	5214	70.3	1270	17.1	936	12.6

Abbreviations - *IMD* Indices of Multiple Deprivation

Comorbidity was over twice as prevalent in lung cancer patients than in patients with HL: 67% of lung cancer patients had one or more comorbidities versus almost 30% of HL patients. Similar patterns in comorbidity prevalence were seen in males and females. The prevalence of either single or multiple comorbidity rose with increasing age. Single comorbidity was more common than multiple comorbidity in the younger age groups, whereas in the older patients the opposite was observed. For example, approximately 29.2% of lung cancer patients aged 15–29 years had one comorbidity and 3.4% had multiple comorbidities, while in lung cancer patients aged 75–90 years the percentage of patients with one comorbidity or with multiple comorbidities were 26.9 and 49.9%, respectively.

The prevalence of multiple comorbidity increased with deprivation level in colon, rectum and lung cancer patients, but there was no pattern with deprivation in HL patients or in the prevalence of one comorbidity. For example, from 24.7 to 25.7% of rectal cancer patients had one comorbidity, while 17.7 to 27.6% of patients had multiple comorbidities.

### Crude and adjusted prevalence of comorbidities at the time of cancer diagnosis

**Fig. 1:**
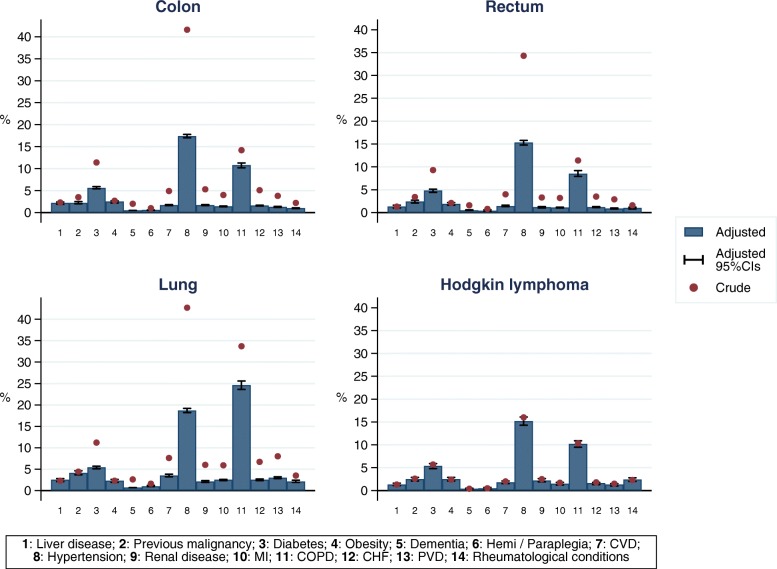
Crude and adjusted prevalence (%) of fourteen comorbidities among cancer patients in England, by cancer

Across all cancer patient cohorts, hypertension, COPD, diabetes, CVD, CHF and PVD were among the most commonly recorded comorbid conditions. Adjusting for age and sex strongly impacted the prevalence of some comorbid conditions in colon, rectum and lung cancer patients (Fig. 1). The three most prevalent comorbidities in all four cancer patient cohorts were hypertension, COPD and diabetes. The adjusted prevalence of hypertension and of diabetes was similar among patients in each of the four cohorts (approximately 15–20% of patients had hypertension while approximately 5% of patients had diabetes). However, the adjusted prevalence of COPD was markedly higher in patients with lung cancer: approximately 25% of lung cancer patients had COPD versus 10% of patients in the other patient cohorts. Similarly, in comparison between the four cohorts, the prevalence of several other conditions (CVD, CHF, PVD or previous malignancy) was highest among the lung cancer patients.

### Combinations of multiple comorbidity

**Fig. 2:**
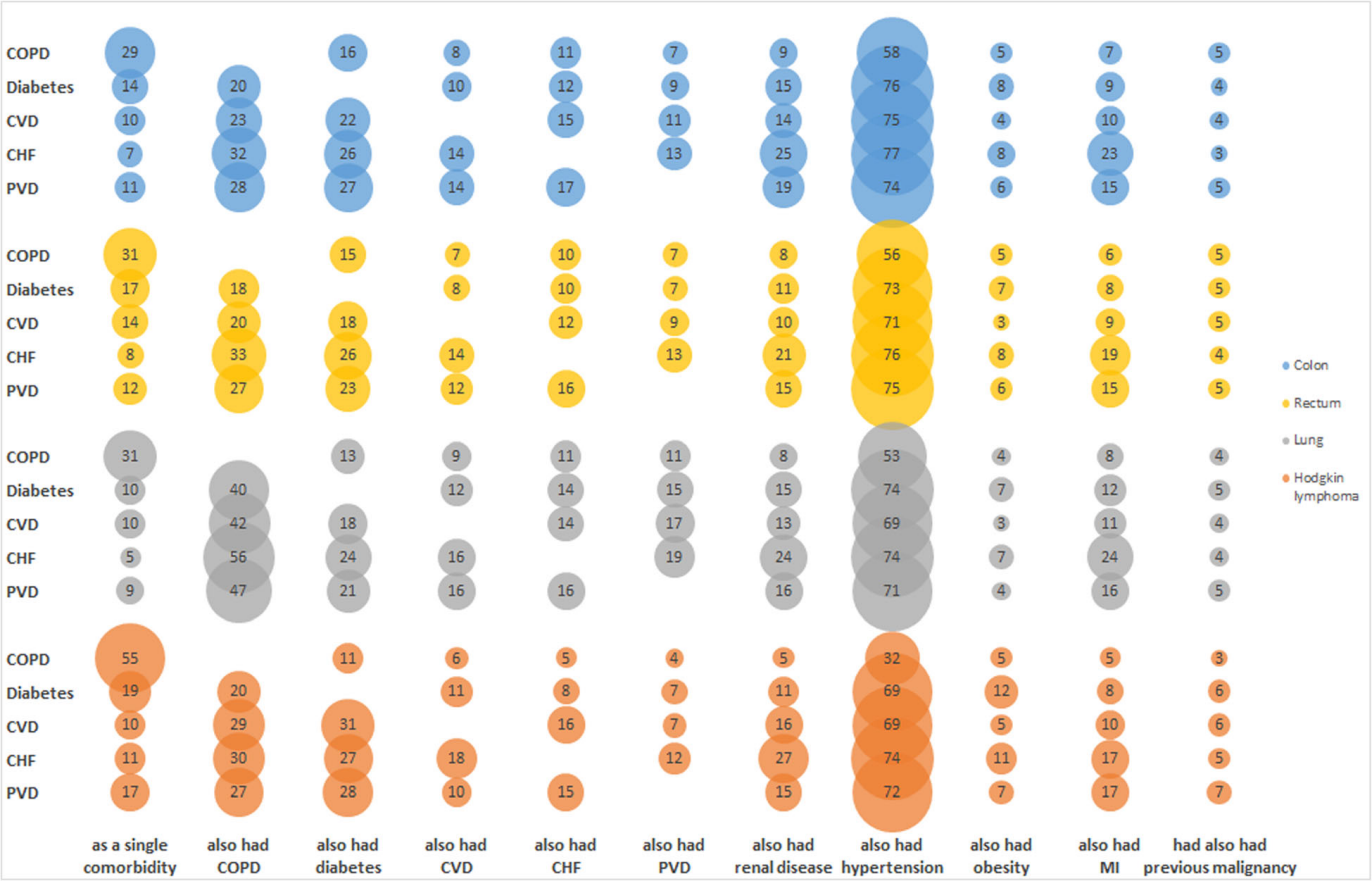
Relative frequency (%) of five common conditions as a single comorbidity or with another comorbidity, by cancer

The relative frequency (%) in which five of the most common conditions (COPD, diabetes, CVD, CHF and PVD) are present either as a single comorbidity or in combination with ten other common comorbid conditions is shown in Fig. 2. For a given cancer (identified by colour), the denominator is the number of patients with the comorbid condition, as represented on the y-axis, and the numerator is the number of those patients who had the condition as a single comorbidity or who had another condition, as depicted by the x-axis. Patients with two or more of the x-axis conditions are represented in the numerator for each condition.

Approximately one third of colorectal and lung cancer patients with COPD, and over half of HL patients with COPD, had this condition as a single comorbidity. By comparison, under one fifth of patients with diabetes, CVD, CHF and PVD had these conditions as a single comorbidity. CHF was the condition least frequently observed as a single comorbidity across all four cancer sites (89% or more of patients with CHF had additional comorbidities).

Hypertension was the condition most commonly present with each of comorbidities for which cross tabulations were investigated. In each of the cancer cohorts, approximately three-quarters of patients with CHF, and a similar proportion with CVD, also had hypertension. COPD was most commonly seen in combination with diabetes, CVD, CHF or PVD in lung cancer patients: while over 50% of lung cancer patients with CHF also had COPD, around one third of patients with HL, colon or rectal cancers with CHF also had COPD.

### Multivariate analysis

The odds ratios derived from logistic regression of each comorbid condition being present at the time of cancer diagnosis, by cancer site, for females relative to males, age (relative to age 70 in colon, rectal and lung cancer patients, and relative to age 45 in HL patients) and increasing deprivation, adjusted for the other listed variables, are shown in Table 2. Analyses conducted for patients with HL were restricted to the comorbidities of diabetes, hypertension and COPD, as the prevalence counts of the other conditions did not adhere to the minimum of ten EPV required for the analyses.

**Table 2:** Odds ratios of condition being present, by cancer (adjusted for other listed variables)

	Liver disease	Previous malignancy	Diabetes	Obesity	Dementia	Hemi- or paraplegia	CVD	Hypertension	Renal disease	MI	COPD	CHF	PVD	Rheum. conditions
	OR (95% CIs)													
	**Colon cancer**													
**Sex**														
Male [REF]	1.00	1.00	1.00	1.00	1.00	1.00	1.00	1.00	1.00	1.00	1.00	1.00	1.00	1.00
Female	0.99 (0.91, 1.07)	1.23 (1.16, 1.32)	0.72 (0.69, 0.75)	0.94 (0.87, 1.01)	1.19 (1.09, 1.30)	0.70 (0.62, 0.79)	0.80 (0.75, 0.85)	0.90 (0.87, 0.92)	0.75 (0.71, 0.80)	0.48 (0.45, 0.51)	1.05 (1.01, 1.09)	0.66 (0.63, 0.70)	0.45 (0.42, 0.48)	2.16 (1.98, 2.36)
**Age at cancer diagnosis (years)**														
45	1.18 (1.13, 1.23)	0.40 (0.39, 0.41)	0.27 (0.25, 0.29)	0.79 (0.76, 0.82)	0.02 (0.02, 0.02)	0.39 (0.38, 0.39)	0.13 (0.13, 0.13)	0.14 (0.12, 0.16)	0.15 (0.15, 0.15)	0.12 (0.11, 0.12)	0.55 (0.49, 0.62)	0.12 (0.12, 0.12)	0.12 (0.12, 0.13)	0.22 (0.22, 0.22)
60	1.13 (1.08, 1.17)	0.74 (0.70, 0.78)	0.64 (0.55, 0.73)	1.00 (0.95, 1.05)	0.18 (0.17, 0.18)	0.65 (0.65, 0.66)	0.46 (0.45, 0.47)	0.51 (0.33, 0.80)	0.41 (0.40, 0.42)	0.52 (0.50, 0.54)	0.72 (0.62, 0.83)	0.45 (0.43, 0.46)	0.43 (0.41, 0.44)	0.54 (0.53, 0.55)
70 [REF]	1.00	1.00	1.00	1.00	1.00	1.00	1.00	1.00	1.00	1.00	1.00	1.00	1.00	1.00
80	1.01 (0.98, 1.05)	1.05 (0.97, 1.12)	1.31 (1.00, 1.71)	0.74 (0.72, 0.77)	4.68 (4.49, 4.89)	1.49 (1.46, 1.53)	2.04 (1.80, 2.32)	1.88 (0.68, 5.21)	2.64 (2.30, 3.03)	1.60 (1.42, 1.80)	1.38 (1.05, 1.80)	2.43 (2.10, 2.80)	1.88 (1.66, 2.13)	1.64 (1.58, 1.71)
90	0.91 (0.88, 0.94)	0.71 (0.68, 0.75)	0.82 (0.69, 0.98)	0.17 (0.17, 0.17)	13.95 (12.35, 15.75)	1.42 (1.39, 1.45)	3.27 (2.69, 3.99)	2.13 (0.72, 6.26)	4.35 (3.50, 5.40)	2.26 (1.92, 2.65)	1.04 (0.84, 1.29)	4.72 (3.63, 6.13)	1.70 (1.52, 1.91)	1.31 (1.27, 1.36)
**Deprivation group**														
Least deprived [REF]	1.00	1.00	1.00	1.00	1.00	1.00	1.00	1.00	1.00	1.00	1.00	1.00	1.00	1.00
2	1.10 (0.96, 1.25)	1.03 (0.93, 1.14)	1.12 (1.05, 1.19)	1.09 (0.95, 1.24)	1.19 (1.03, 1.37)	1.25 (1.02, 1.54)	1.11 (1.01, 1.21)	1.08 (1.03, 1.12)	1.20 (1.09, 1.31)	1.09 (0.99, 1.21)	1.12 (1.06, 1.18)	1.07 (0.98, 1.17)	1.11 (1.00, 1.24)	1.08 (0.96, 1.23)
3	1.13 (0.99, 1.29)	0.99 (0.89, 1.09)	1.25 (1.18, 1.33)	1.56 (1.38, 1.76)	1.29 (1.11, 1.48)	1.47 (1.20, 1.79)	1.20 (1.09, 1.31)	1.18 (1.13, 1.23)	1.34 (1.23, 1.47)	1.21 (1.10, 1.34)	1.24 (1.18, 1.32)	1.16 (1.06, 1.27)	1.26 (1.14, 1.40)	0.95 (0.84, 1.08)
4	1.38 (1.22, 1.58)	1.03 (0.93, 1.15)	1.48 (1.39, 1.58)	1.73 (1.53, 1.95)	1.42 (1.23, 1.63)	1.47 (1.20, 1.80)	1.35 (1.23, 1.47)	1.32 (1.27, 1.38)	1.55 (1.42, 1.69)	1.33 (1.21, 1.47)	1.56 (1.47, 1.65)	1.38 (1.26, 1.50)	1.38 (1.25, 1.53)	1.00 (0.88, 1.14)
Most deprived	1.50 (1.31, 1.71)	1.13 (1.02, 1.26)	1.75 (1.64, 1.87)	1.91 (1.68, 2.16)	1.70 (1.47, 1.97)	2.29 (1.88, 2.79)	1.60 (1.46, 1.76)	1.54 (1.47, 1.61)	1.83 (1.67, 2.01)	1.55 (1.40, 1.72)	2.01 (1.89, 2.12)	1.67 (1.52, 1.83)	1.59 (1.42, 1.76)	0.99 (0.86, 1.14)
	**Rectal cancer**													
**Sex**														
Male [REF]	1.00	1.00	1.00	1.00	1.00	1.00	1.00	1.00	1.00	1.00	1.00	1.00	1.00	1.00
Female	1.05 (0.91, 1.22)	1.34 (1.23, 1.47)	0.80 (0.75, 0.85)	1.17 (1.04, 1.32)	1.29 (1.13, 1.48)	0.66 (0.54, 0.81)	0.75 (0.69, 0.82)	0.95 (0.91, 0.99)	0.73 (0.66, 0.80)	0.52 (0.46, 0.58)	1.03 (0.97, 1.09)	0.72 (0.65, 0.79)	0.39 (0.34, 0.44)	1.97 (1.73, 2.25)
**Age at cancer diagnosis (years)**														
45	0.93 (0.91, 0.95)	0.42 (0.41, 0.43)	0.27 (0.26, 0.29)	0.69 (0.68, 0.71)	0.06 (0.06, 0.06)	0.39 (0.39, 0.40)	0.12 (0.12, 0.12)	0.13 (0.12, 0.15)	0.12 (0.12, 0.13)	0.11 (0.11, 0.11)	0.47 (0.43, 0.51)	0.12 (0.12, 0.12)	0.07 (0.07, 0.07)	0.30 (0.29, 0.30)
60	1.00 (0.98, 1.02)	0.68 (0.65, 0.71)	0.60 (0.54, 0.66)	0.95 (0.92, 0.98)	0.21 (0.21, 0.21)	0.60 (0.60, 0.60)	0.46 (0.45, 0.47)	0.49 (0.34, 0.71)	0.37 (0.36, 0.37)	0.49 (0.48, 0.51)	0.66 (0.59, 0.74)	0.44 (0.43, 0.44)	0.40 (0.39, 0.41)	0.58 (0.57, 0.59)
70 [REF]	1.00	1.00	1.00	1.00	1.00	1.00	1.00	1.00	1.00	1.00	1.00	1.00	1.00	1.00
80	0.98 (0.96, 1.00)	1.22 (1.13, 1.32)	1.37 (1.11, 1.71)	0.73 (0.71, 0.74)	5.57 (5.32, 5.84)	1.83 (1.81, 1.86)	2.14 (1.92, 2.39)	1.74 (0.71, 4.22)	2.61 (2.35, 2.90)	1.63 (1.48, 1.80)	1.46 (1.17, 1.83)	2.46 (2.21, 2.74)	1.80 (1.62, 1.99)	1.65 (1.60, 1.70)
90	1.11 (1.09, 1.13)	0.96 (0.90, 1.02)	0.83 (0.72, 0.95)	0.17 (0.17, 0.17)	13.80 (12.36, 15.40)	1.82 (1.79, 1.85)	4.07 (3.35, 4.94)	2.51 (0.86, 7.29)	5.14 (4.22, 6.26)	2.06 (1.82, 2.32)	1.29 (1.06, 1.58)	4.96 (4.04, 6.10)	2.24 (1.97, 2.54)	1.91 (1.84, 1.99)
**Deprivation group**														
Least deprived [REF]	1.00	1.00	1.00	1.00	1.00	1.00	1.00	1.00	1.00	1.00	1.00	1.00	1.00	1.00
2	1.00 (0.77, 1.28)	1.08 (0.94, 1.24)	1.14 (1.04, 1.26)	1.28 (1.06, 1.56)	1.10 (0.89, 1.37)	1.70 (1.20, 2.40)	1.13 (0.98, 1.30)	1.04 (0.99, 1.10)	1.06 (0.91, 1.24)	1.06 (0.91, 1.24)	1.14 (1.04, 1.25)	1.11 (0.95, 1.29)	1.06 (0.90, 1.24)	1.09 (0.89, 1.34)
3	1.50 (1.19, 1.89)	0.91 (0.79, 1.05)	1.29 (1.18, 1.42)	1.27 (1.04, 1.54)	1.21 (0.98, 1.50)	2.13 (1.52, 2.98)	1.39 (1.21, 1.60)	1.14 (1.07, 1.20)	1.26 (1.08, 1.46)	1.17 (1.00, 1.36)	1.36 (1.25, 1.49)	1.23 (1.06, 1.42)	1.11 (0.94, 1.30)	1.13 (0.92, 1.39)
4	1.33 (1.05, 1.69)	1.01 (0.88, 1.17)	1.60 (1.46, 1.75)	1.63 (1.35, 1.97)	1.30 (1.05, 1.60)	2.23 (1.59, 3.12)	1.51 (1.32, 1.74)	1.26 (1.19, 1.34)	1.34 (1.15, 1.55)	1.33 (1.14, 1.54)	1.62 (1.49, 1.76)	1.25 (1.07, 1.44)	1.34 (1.14, 1.57)	1.12 (0.91, 1.38)
Most deprived	1.77 (1.39, 2.24)	1.14 (0.99, 1.33)	1.80 (1.63, 1.97)	1.80 (1.48, 2.18)	1.63 (1.31, 2.02)	2.66 (1.89, 3.74)	1.78 (1.54, 2.05)	1.48 (1.39, 1.57)	1.57 (1.34, 1.83)	1.52 (1.30, 1.77)	2.25 (2.07, 2.46)	1.65 (1.42, 1.91)	1.59 (1.36, 1.87)	1.24 (1.00, 1.54)
	**Lung cancer**													
**Sex**														
Male [REF]	1.00	1.00	1.00	1.00	1.00	1.00	1.00	1.00	1.00	1.00	1.00	1.00	1.00	1.00
Female	0.87 (0.82, 0.93)	1.11 (1.06, 1.16)	0.75 (0.73, 0.78)	1.12 (1.05, 1.20)	1.22 (1.15, 1.30)	0.81 (0.75, 0.88)	0.83 (0.80, 0.87)	0.98 (0.96, 1.00)	0.72 (0.69, 0.75)	0.60 (0.58, 0.63)	1.09 (1.07, 1.11)	0.76 (0.73, 0.80)	0.50 (0.48, 0.52)	1.98 (1.87, 2.09)
**Age at cancer diagnosis (years)**														
45	1.42 (1.35, 1.49)	0.83 (0.76, 0.89)	0.24 (0.23, 0.26)	0.70 (0.68, 0.71)	0.04 (0.04, 0.04)	0.43 (0.43, 0.43)	0.22 (0.21, 0.23)	0.12 (0.11, 0.14)	0.13 (0.13, 0.13)	0.19 (0.19, 0.20)	0.39 (0.31, 0.50)	0.16 (0.16, 0.17)	0.10 (0.09, 0.10)	0.29 (0.28, 0.29)
60	1.35 (1.29, 1.42)	0.93 (0.85, 1.01)	0.60 (0.52, 0.69)	0.97 (0.93, 1.00)	0.28 (0.28, 0.28)	0.75 (0.74, 0.76)	0.55 (0.52, 0.59)	0.49 (0.30, 0.80)	0.38 (0.36, 0.39)	0.62 (0.58, 0.66)	0.69 (0.47, 1.03)	0.47 (0.45, 0.50)	0.47 (0.43, 0.51)	0.70 (0.68, 0.73)
70 [REF]	1.00	1.00	1.00	1.00	1.00	1.00	1.00	1.00	1.00	1.00	1.00	1.00	1.00	1.00
80	0.80 (0.78, 0.82)	1.02 (0.92, 1.12)	1.24 (0.95, 1.63)	0.67 (0.65, 0.69)	4.37 (4.13, 4.63)	1.10 (1.07, 1.12)	1.56 (1.31, 1.85)	1.59 (0.57, 4.41)	2.48 (2.07, 2.97)	1.38 (1.19, 1.61)	1.10 (0.63, 1.94)	1.93 (1.62, 2.29)	1.47 (1.15, 1.87)	1.07 (1.02, 1.13)
90	0.54 (0.53, 0.55)	0.76 (0.71, 0.82)	0.82 (0.68, 0.99)	0.18 (0.18, 0.18)	13.22 (11.20, 15.60)	1.23 (1.20, 1.27)	2.26 (1.77, 2.88)	1.70 (0.59, 4.85)	4.16 (3.13, 5.53)	1.51 (1.28, 1.78)	0.80 (0.51, 1.24)	3.55 (2.63, 4.79)	1.08 (0.90, 1.30)	0.92 (0.88, 0.96)
**Deprivation group**														
Least deprived [REF]	1.00	1.00	1.00	1.00	1.00	1.00	1.00	1.00	1.00	1.00	1.00	1.00	1.00	1.00
2	1.28 (1.13, 1.46)	0.95 (0.87, 1.03)	1.10 (1.04, 1.16)	1.19 (1.05, 1.36)	1.13 (1.01, 1.27)	1.38 (1.17, 1.62)	1.09 (1.01, 1.17)	1.02 (0.98, 1.05)	1.07 (1.00, 1.16)	1.08 (1.00, 1.17)	1.17 (1.12, 1.22)	1.11 (1.03, 1.19)	1.12 (1.05, 1.20)	1.09 (0.99, 1.20)
3	1.29 (1.14, 1.47)	0.89 (0.82, 0.96)	1.09 (1.03, 1.15)	1.27 (1.12, 1.45)	1.21 (1.09, 1.36)	1.50 (1.28, 1.76)	1.20 (1.12, 1.28)	1.04 (1.01, 1.08)	1.15 (1.07, 1.24)	1.16 (1.08, 1.25)	1.36 (1.31, 1.41)	1.15 (1.07, 1.23)	1.14 (1.07, 1.21)	1.04 (0.95, 1.14)
4	1.44 (1.27, 1.62)	0.84 (0.78, 0.91)	1.25 (1.18, 1.32)	1.51 (1.34, 1.70)	1.38 (1.24, 1.53)	1.76 (1.51, 2.04)	1.36 (1.27, 1.45)	1.13 (1.09, 1.17)	1.21 (1.13, 1.30)	1.28 (1.19, 1.38)	1.59 (1.53, 1.65)	1.30 (1.22, 1.39)	1.17 (1.10, 1.25)	1.08 (0.99, 1.18)
Most deprived	1.66 (1.48, 1.87)	0.88 (0.81, 0.95)	1.29 (1.23, 1.36)	1.69 (1.50, 1.90)	1.52 (1.37, 1.69)	2.17 (1.88, 2.52)	1.45 (1.36, 1.54)	1.22 (1.18, 1.27)	1.33 (1.24, 1.43)	1.33 (1.24, 1.43)	1.96 (1.89, 2.03)	1.35 (1.26, 1.44)	1.32 (1.24, 1.41)	1.05 (0.96, 1.14)
	**Hodgkin lymphoma**													
**Sex**														
Male [REF]	–	–	1.00	–	–	–	–	1.00	–	–	1.00	–	–	–
Female	–	–	0.62 (0.50, 0.77)	–	–	–	–	0.88 (0.76, 1.02)	–	–	1.05 (0.90, 1.23)	–	–	–
**Age at cancer diagnosis (years)**														
45 [REF]	–	–	1.00	–	–	–	–	1.00	–	–	1.00	–	–	–
60	–	–	3.36 (2.89, 3.91)	–	–	–	–	4.46 (3.00, 6.63)	–	–	1.68 (1.41, 2.00)	–	–	–
70	–	–	5.03 (4.04, 6.27)	–	–	–	–	8.78 (4.57, 16.86)	–	–	2.48 (1.94, 3.18)	–	–	–
80	–	–	5.74 (4.49, 7.33)	–	–	–	–	13.86 (5.84, 32.91)	–	–	2.62 (2.02, 3.39)	–	–	–
90	–	–	5.17 (4.13, 6.47)	–	–	–	–	18.13 (6.70, 49.03)	–	–	1.43 (1.23, 1.66)	–	–	–
**Deprivation group**														
Least deprived [REF]	–	–	1.00	–	–	–	–	1.00	–	–	1.00	–	–	–
2	–	–	1.19 (0.83, 1.72)	–	–	–	–	1.26 (1.00, 1.60)	–	–	1.17 (0.90, 1.52)	–	–	–
3	–	–	1.48 (1.03, 2.11)	–	–	–	–	1.43 (1.14, 1.81)	–	–	1.18 (0.91, 1.54)	–	–	–
4	–	–	1.89 (1.34, 2.67)	–	–	–	–	1.48 (1.18, 1.87)	–	–	1.37 (1.07, 1.77)	–	–	–
Most deprived	–	–	2.39 (1.69, 3.37)	–	–	–	–	1.96 (1.55, 2.48)	–	–	1.86 (1.45, 2.38)	–	–	–

Abbreviations - *CI* confidence intervals, *CVD* Cerebrovascular disease, *MI* Myocardial infarction, *COPD* Chronic obstructive pulmonary disease, *CHF* Congestive heart failure, *PVD* Pheripheral vascular disease, *REF* reference, *Rheum.* Rheumatological

Female patients with colon, rectal or lung cancer had up to 29% increased adjusted odds of having dementia (rectal cancer: OR 1.29; 95%CI 1.13, 1.48), up to 34% increased adjusted odds of having a previous malignancy (rectal cancer: OR 1.34; 1.23, 1.47) and approximately twice the adjusted odds of having rheumatological conditions (colon cancer: OR 2.16; 1.98, 2.36) compared to male patients. Conversely, compared with male patients in their respective cohort, females had significantly reduced adjusted odds of having diabetes, hemiplegia or paraplegia, CVD, renal disease, MI, CHF or PVD. Across all four cancer cohorts, female patients had up to 38% reduced odds of having diabetes (HL: OR 0.62; 95%CI 0.50, 0.77).

The adjusted odds of dementia, CVD, hypertension, renal disease, MI and CHF being present at diagnosis consistently increased with age. For example, with 70-year old patients as the reference, colon cancer patients aged 45 had 87% reduced adjusted odds of CVD (OR 0.13; 0.13, 0.13) and 88% reduced adjusted odds of CHF (OR 0.12; 0.12, 0.12), while 90-year old patients had over three times the adjusted odds of CVD (OR 3.27; 2.69, 3.99) and over four times the adjusted odds of CHF (OR 4.72; 3.63, 6.13). There was no trend with age in colon, rectal or lung cancer patients for liver disease, having had a previous malignancy, diabetes or obesity. In lung cancer patients, no trend was observed with age for having COPD.

For at least eleven of the fourteen conditions, the adjusted odds of having the comorbid condition increased with the level of deprivation in colon, rectal or lung cancer patients. Obesity, dementia, hemiplegia, CVD, hypertension, renal disease, MI, COPD, CHF and PVD were associated with deprivation level in all three cancer cohorts. For example, the most deprived groups of lung cancer and colon cancer patients had approximately twice the adjusted odds of having COPD compared with the least deprived groups (OR 1.96; 1.89, 2.03 and OR 2.01; 1.89, 2.12 in the most deprived patients with lung or colon cancer, respectively). No trend with deprivation was seen with rheumatological conditions or with having a previous malignancy.

### Probability of having single or multiple comorbidity at the time of cancer diagnosis

**Fig. 3:**
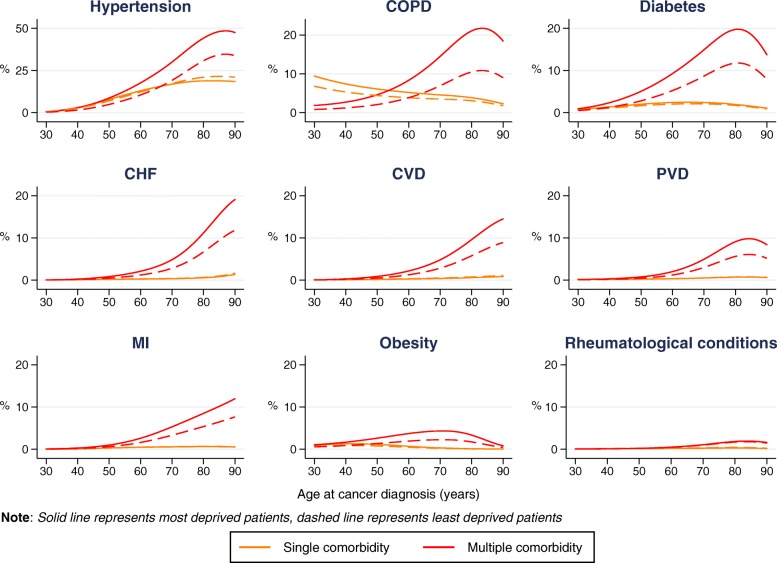
Probability (%) of condition present as single or multiple comorbidity, by deprivation group (colon cancer)

The graphs depicted in Fig. 3 show the adjusted probability of patients having one of the nine most common comorbid conditions recorded (hypertension, COPD, diabetes, CHF, CVD, PVD, MI, obesity or rheumatological conditions) at the time of colon cancer diagnosis, either as a single comorbidity, or as one of multiple comorbidities, according to age at cancer diagnosis and deprivation group (the least and most deprived groups), as derived from multinomial logistic regression.

With the exception of COPD, there was little difference between the most and least deprived groups in the probability of having each of the conditions as a single comorbidity. Among those patients with COPD as a single comorbidity, the difference in probability between the most and least deprived groups decreased with age. The most deprived patients had a higher probability of having each of the conditions as one of multiple comorbidities compared with the least deprived group, with one exception (rheumatological conditions). Generally, the difference in probability between the two deprivation groups was greatest in older age: it peaked at approximately 80 years for hypertension, COPD, diabetes, PVD and obesity, while in patients with CHF, CVD, and MI the difference continued to increase with age. Having rheumatological conditions was not associated with increasing age or deprivation level.

Similar patterns in the probability of having a comorbid condition according to deprivation group were observed for patients with rectal or lung cancers (Additional files 2 and 3).

## Discussion

Our study is, to our knowledge, the first large-scale, population-based study describing comorbidity prevalence in cancer patient populations. Up to two-thirds of patients had at least one long-term health condition at the time of their cancer diagnosis, and around half of these comorbid cancer patients had multiple long-term conditions. There was evidence that many of the comorbid conditions we investigated were associated with socio-economic deprivation, and the most deprived groups of patients had a higher probability of having multiple comorbidities compared with the less deprived groups.

The choice of cancer sites we studied was based on aetiology of the cancer: three of the cancer sites (colon, rectum and lung) were associated with environmental risk factors including tobacco smoking [22, 23], alcohol use and diet [24, 25]. Furthermore, tobacco smoking is associated with certain conditions, such as COPD [26–28] and Type 2 diabetes [29, 30], and is also associated with socioeconomic position [31]. HL is linked to infection rather than environmental factors [22].

Hypertension, COPD and diabetes were the three most prevalent comorbidities in all four cancer patient cohorts, with a higher prevalence in the most deprived patients. The odds of having COPD from being in the most deprived group of lung cancer patients (compared with being in the least deprived group – the ‘deprivation gap’) was 10% more than the deprivation gap in the adjusted odds of having COPD in the Hodgkin lymphoma patients. This may be reflective of the role of smoking in the aetiology of both lung cancer and COPD, and the higher prevalence of smoking in the more deprived population. The association between smoking status and deprivation is not quantifiable in the cancer patient cohorts as we did not have information on smoking prevalence.

Similar work using administrative data to describe comorbidity in cancer populations has been undertaken in New Zealand [32] and in Spain [33]. In the study of patients diagnosed with colon, rectal, breast, ovarian, uterine, stomach, liver, renal or bladder cancers in New Zealand (*N* = 14,096), commonly diagnosed comorbidities among colon and rectal cancer patients were hypertension, cardiac conditions and diabetes. In the Spanish cohort of colorectal cancer patients from the cancer registries of Girona and Granada (*N* = 1061), diabetes, COPD and CHF were the most common comorbidities. Comparing our study with the study in New Zealand, there were similarities among colon cancer patients in the age-sex adjusted prevalence of hypertension, while diabetes prevalence was higher in New Zealand. The adjusted prevalence of hypertension was 16.6%, uncomplicated diabetes was 5.9% and diabetes with complications was 5.0% among patients in New Zealand, while in our study the adjusted prevalence of hypertension was 17.4% and diabetes (with and without complications) was 5.7%. This supports our earlier assumption that less severe diabetes may be underreported in hospital admissions records. Given the ‘gatekeeper’ structure and functioning of the healthcare system in the UK [34] and the focus on managing diabetes within primary care [35], cases of diabetes recorded in hospital admissions are possibly those that are not controlled within available primary care resources [36] or present with complications. The Spanish study reported the crude prevalence of conditions among colorectal cancer patients, which were generally higher than the crude prevalence of conditions observed in our study. Diabetes was prevalent in 23.6% of colorectal cancer patients in this study, while in our study the crude prevalence of diabetes was 11.4% or 9.4% among colon or rectal cancer patients, respectively. Nonetheless, there was consistency between our study and both of these other studies in terms of common comorbid conditions among the patient cohorts.

In our study, approximately 13% of the HL cohort, over 21% of the colorectal cancer cohorts and over 39% of the lung cancer cohort had multiple comorbidities, while from 17 to 28% of patients in each cohort had a single comorbidity at the time of their cancer diagnosis. These findings are important given the impact comorbidity may have on cancer care, particularly where care is provided within the constraints of healthcare guidelines that are not designed for the simultaneous management of two or more chronic conditions or morbidities (i.e. “multimorbidity”). Scientific studies indicate that multimorbidity is regularly observed in the population [37–39] and poses a challenge to health care systems, particularly those geared towards single disease management [5, 40, 41]. Clinical guidelines in the United Kingdom are not accommodating to the cumulative impact of treatment recommendations on those with multiple morbidities, and do not facilitate a comparison of potential benefits or risks [42]. Patients with multiple chronic conditions have higher rates of healthcare consultations than those without [38, 43, 44]. Managing and treating comorbid conditions places an additional economic burden on healthcare systems. In one study of the costs per capita of several comorbid conditions, renal disease was identified as one of the most costly conditions to manage among cancer patients (approximately 174% of the costs of the cancer), while the cost of diabetes or heart disease was substantially lower (approximately 20% or 6% of cancer costs, respectively) [45]. The increase in costs also depends on the number and combination of comorbid conditions: among the cancer patients with diabetes in our study, between 10 and 15% of these patients also had renal disease.

In cancer patients, the presence of comorbidity can be influential on cancer management and therapeutic options. Patients with comorbidity may be less likely than those without comorbidity to receive curative treatment [3]. Treatment decisions made by clinicians may be weighted by the type and severity of comorbidity, for example, CHF has been reported to influence receipt of surgery for non-small cell lung cancer [46], receipt of adjuvant chemotherapy for colon cancer [47] and receipt of any treatment for prostate cancer [48]. The presence of COPD influenced receipt of surgical treatment in non-small cell lung cancer patients [46] and adjuvant therapy in colon cancer patients [47]. However, there is also evidence that comorbid patients who receive treatment have better prognosis for survival than those who do not receive treatment, as shown with the receipt of adjuvant therapy for colon cancer [47, 49]. Moreover, older cancer patients and patients with comorbidity have historically been under-represented in cancer clinical trials. This limits the applicability of cancer clinical trial results to a younger and healthier cohort of patients than clinicians are actually treating, meaning that while there is evidence suggesting that patients with comorbidity as a group are not receiving optimal cancer treatment, specific information required for clinical decision-making is often lacking [50]. We found a non-negligible increase in the prevalence of comorbidities when we included diagnoses in the six-months prior to cancer diagnoses. While some of these conditions may have arisen in these months because of the cancer, their presence will be as relevant when considering treatment, irrespective of the timing of their diagnosis.

Our study showed socio-economic position to be an important factor associated with having one or more comorbid conditions at the time of cancer diagnosis, with comorbidity prevalence increasing with deprivation. It is possible that mechanisms within clinical guidelines and decision-making that lead to non-treatment of cancer patients with comorbidity disproportionately impact the more deprived patients. An existence of socio-economic inequalities in receipt of treatment has been identified [51, 52]. Reviewing the treatment process of cancer patients with comorbidity may therefore have a beneficial effect in reducing the socioeconomic inequalities in receipt of cancer treatment. Moreover, because cancer data contains mainly cancer-related outcomes, how the cancer and related treatments impact patient comorbidity and prognosis is not well known [3]. Having the resources and guidelines within which to manage patient comorbid conditions robustly during cancer treatment is one strategy for mitigating the risk of adverse patient outcomes occurring from comorbid disease. In England, socio-economic inequalities in cancer survival have narrowed little, despite the implementation of government strategies that intended to reduce these inequalities [53]. Focusing on the management of comorbidity in cancer patients could be one potential pathway to addressing socio-economic inequalities in cancer outcomes.

There are a variety of metrics of comorbidity in the scientific literature that are used to study the relationship between comorbidity on cancer outcomes, although no consensus has been reached on a gold standard measure of comorbidity within the context of cancer [54]. Many of the approaches provide a summary measure of the patient’s comorbid conditions and the severity of these conditions. However, the prognostic impact of comorbidity can depend on the type and stage of the cancer [55]. In addition, the presence of comorbidity - particularly certain comorbid conditions - adds complexity to the provision of treatment for cancer. When investigating the relationship between comorbidity and cancer outcomes, a more granular approach investigating specific comorbid conditions in turn, rather than using a summary measure of comorbidity, could be more appropriate and insightful.

We acknowledge potential limitations in this study. We capture comorbidity information based on diagnoses of health conditions recorded during hospital admission(s) prior to cancer diagnosis, and are therefore reliant on patients requiring hospital-based medical attention for their health condition(s) in order to obtain this information. The potential for measurement error from the information recorded in the diagnostic fields of hospital admissions records should also be acknowledged. However, we assume that the more severe conditions are likely to be captured within the diagnostic fields. Underreporting may occur in less severe conditions, such as obesity, that are unlikely to be the primary reason for the hospital admission, and may occur more frequently with elderly patients or patients with more severe comorbidities, due to competing demands. Conditions such as less severe type II diabetes are possibly underreported. Further work comparing the prevalence of the conditions we studied in the cancer cohorts with the prevalence of these conditions in the general population in England, as reported in government publications and scientific literature, would be useful step in validating our results.

Our study of over 300,000 patients is one of the largest population-based studies of comorbidity prevalence among cancer patients, and one of the first such studies of patients in England. Using data from well-established sources, we were able to describe the prevalence of fourteen chronic health conditions among these cancer patients, and highlight an association between socio-economic position and prevalence of most of these conditions.

## Conclusion

This study underlines that many comorbid cancer patients are living with multiple comorbidities, and that the most deprived patients carry the greater burden of comorbidity. Healthcare guidelines may not always encompass the simultaneous management of multiple chronic conditions, but guidelines for the management of cancer may need to consider some prominent comorbid conditions. Insight into patterns of cancer comorbidity informs further research into the influence of comorbidity - particularly the influence of specific comorbid conditions - on outcomes following cancer diagnosis, including socio-economic inequalities in receipt of treatment and short-term mortality.

## Supplementary information

**Additional file 1.** Definition of the fourteen conditions, according to ICD-10 code classification. Table of the fourteen conditions and the ICD-10 code groupings used to define them.

**Additional file 2.** Probability (%) of condition present as single or multiple comorbidity, by deprivation group (lung cancer). Additional results in complement to those presented in Fig. 3: graphs representing the probability of having any of nine comorbid conditions in lung cancer patients.

**Additional file 3.** Probability (%) of condition present as single or multiple comorbidity, by deprivation group (rectal cancer). Additional results in complement to those presented in Fig. 3: graphs representing the probability of having any of nine comorbid conditions in rectal cancer patients.

## Data Availability

The data that support the findings of this study are available via application to the Public Health England Office for Data Release, but restrictions apply to the availability of these data.

## Abbreviations

CCI: Charlson Comorbidity Index
CHF: Congestive Heart Failure
CI: Confidence Interval
COPD: Chronic Obstructive Pulmonary Disease
CVD: Cerebrovascular Disease
EPV: Events Per Variable
HES: Hospital Episode Statistics
HL: Hodgkin lymphoma
ICD: International Classification of Diseases and Related Health Problems
MI: Myocardial infarction
OR: Odds Ratio
PVD: Peripheral Vascular Disease
