# Supplementary material for: Comorbidity prevalence among cancer patients: a population-based cohort study of four cancers
Source: BMC Cancer. 2020 Jan 28;20:2. doi: 10.1186/s12885-019-6472-9 (PMC6986047; doi:10.1186/s12885-019-6472-9)
Supplement: Supplementary file 2 — Additional file 2. Probability (%) of condition present as single or multiple comorbidity, by deprivation group (lung cancer). Additional results in complement to those presented in Fig. 3: graphs representing the probability of having any of nine comorbid conditions in lung cancer patients. [file 12885_2019_6472_MOESM2_ESM.pdf]

Additional file 2: Probability (%) of condition present as a single or multiple comorbidity, by deprivation group (**Lung cancer**)

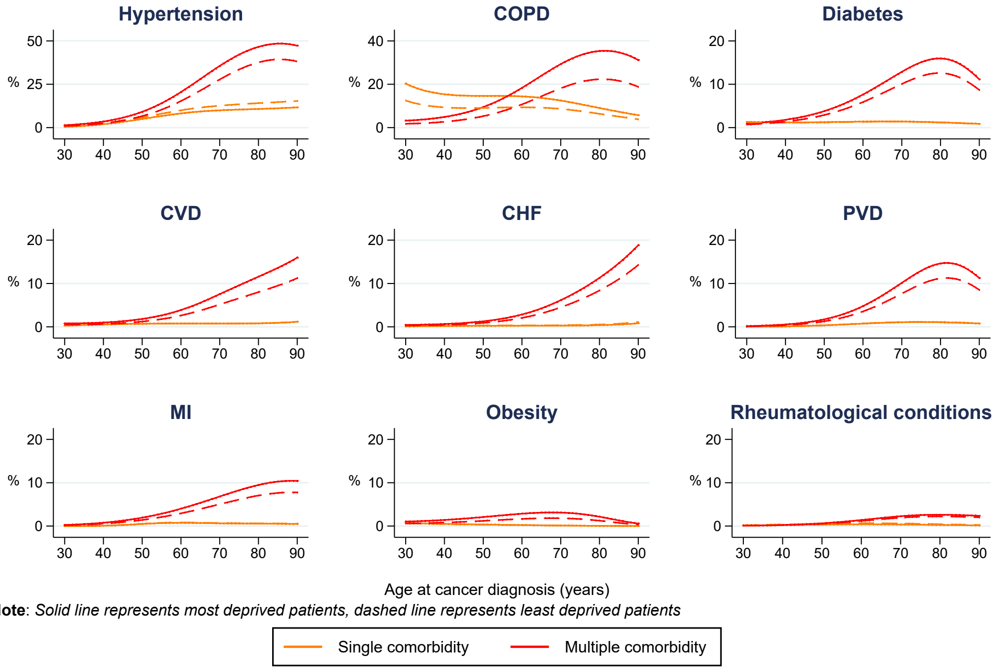

Note: *Solid line represents most deprived patients, dashed line represents least deprived patients*
