# Supplementary material for: Comorbidity prevalence among cancer patients: a population-based cohort study of four cancers
Source: BMC Cancer. 2020 Jan 28;20:2. doi: 10.1186/s12885-019-6472-9 (PMC6986047; doi:10.1186/s12885-019-6472-9)
Supplement: Supplementary file 3 — Additional file 3. Probability (%) of condition present as single or multiple comorbidity, by deprivation group (rectal cancer). Additional results in complement to those presented in Fig. 3: graphs representing the probability of having any of nine comorbid conditions in rectal cancer patients. [file 12885_2019_6472_MOESM3_ESM.pdf]

Additional file 3: Probability (%) of condition present as a single or multiple comorbidity, by deprivation group (**Rectal cancer**)

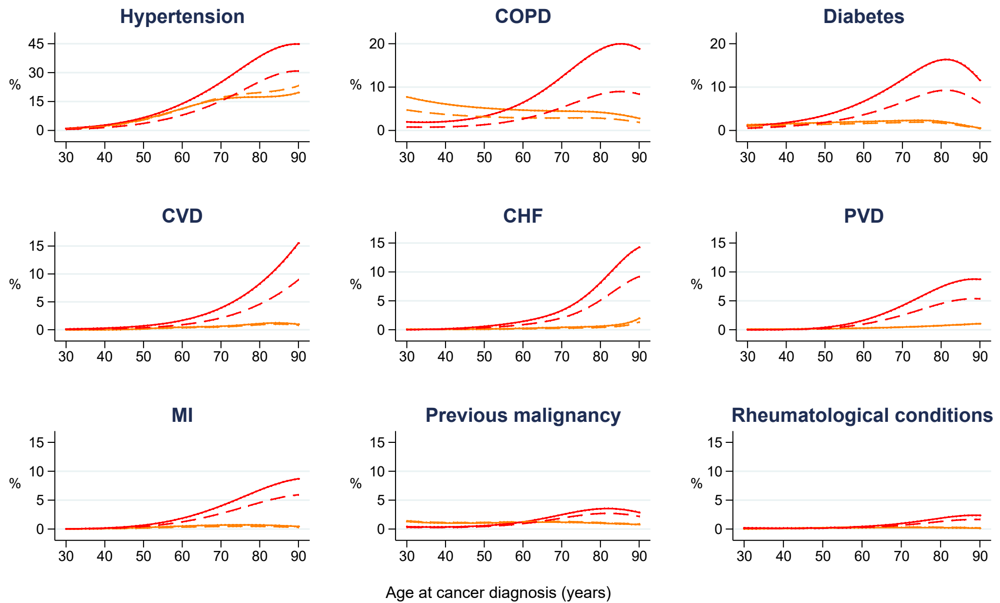

Note: *Solid line represents most deprived patients, dashed line represents least deprived patients*

Single comorbidity — Multiple comorbidity
